# Supplementary material for: Thermal management towards ultra-bright and stable perovskite nanocrystal-based pure red light-emitting diodes
Source: Nat Commun. 2024 Aug 3;15:6561. doi: 10.1038/s41467-024-50634-0 (PMC11297279; doi:10.1038/s41467-024-50634-0)
Supplement: Supplementary file 1 — Supplementary Information [file 41467_2024_50634_MOESM1_ESM.pdf]

Supplementary Information

Thermal Management Towards Ultra-bright and Stable Perovskite Nanocrystal-based Pure Red Light-Emitting Diodes

Hongjin Li[1,2†], Xiaofang Zhu[1,2†], Dingshuo Zhang[1,2], Yun Gao[1,2], Yifeng Feng[1,2], Zichao Ma[1,2], Jingyun Huang[1,2], Haiping He[1,2,3], Zhizhen Ye[1,2,3]*, Xingliang Dai[1,2,3]*

[1] School of Materials Science and Engineering, State Key Laboratory of Silicon and Advanced Semiconductor Materials, Zhejiang University, Hangzhou 310027, P. R. China.

[2] Wenzhou Key Laboratory of Novel Optoelectronic and Nano Materials and Engineering Research Centre of Zhejiang Province, Institute of Wenzhou, Zhejiang University, Wenzhou 325006, P. R. China.

[3] Shanxi-Zheda Institute of Advanced Materials and Chemical Engineering, Taiyuan, 030002 P. R. China.

[†]These authors contributed equally to this work.

*Corresponding authors: Xingliang Dai (shanfeng@zju.edu.cn); Zhizhen Ye (yezz@zju.edu.cn)

Table of contents

Supplementary Fig. 1. Reaction processes between DPPA and precursor solvent.

Supplementary Fig. 2. TEM images.

Supplementary Fig. 3. HAADF-STEM image and corresponding elemental mapping images.

Supplementary Fig. 4. XRD patterns of the pristine-NCs and DPPA-NCs.

Supplementary Fig. 5. HRTEM images.

Supplementary Fig. 6. Optimized crystal structures for the $CsPb(Br/I)_3$ slab models.

Supplementary Fig. 7. Time-resolved photoluminescence decay curves of the NC films.

Supplementary Fig. 8. TA measurements.

Supplementary Fig. 9. Excitation intensity dependence of the PLQYs of the NC solution.

Supplementary Fig. 10. The quantitative 1H NMR spectrum of DPP-NCs.

Supplementary Fig. 11. Thermal stability of the NC films.

Supplementary Fig. 12. CIE coordinate of PeLEDs based on DPPA-NCs.

Supplementary Fig. 13. The flat-band energy level diagram and UPS spectra.

Supplementary Fig. 14. Effect of TMPI on device performance.

Supplementary Fig. 15. Ion migration activation energy measurement.

Supplementary Fig. 16. Infrared-thermal-imaging images of the PeLEDs.

Supplementary Fig. 17. EQE-L curves of DPPA-NC-based PeLEDs on the glass or sapphire substrate.

Supplementary Fig. 18. Statistical distribution histograms of peak EQE and maximum luminance.

Supplementary Fig. 19. EL spectra of the PeLEDs under different current densities.

Supplementary Fig. 20. Pulsed mode operation of PeLED on a sapphire substrate at 50 Hz with a duty cycle of 50%.

Supplementary Table 1. Summary of time-resolved PL exponential fitting parameters for NC solutions.

Supplementary Table 2. Summary of time-resolved PL exponential fitting parameters for NC films.

Supplementary Table 3. Summary of TA spectra fitting parameters.

Supplementary Table 4. Summary of reported red PeLEDs with superior performance.

Supplementary Table 5. The fitting data from the current decay plots.

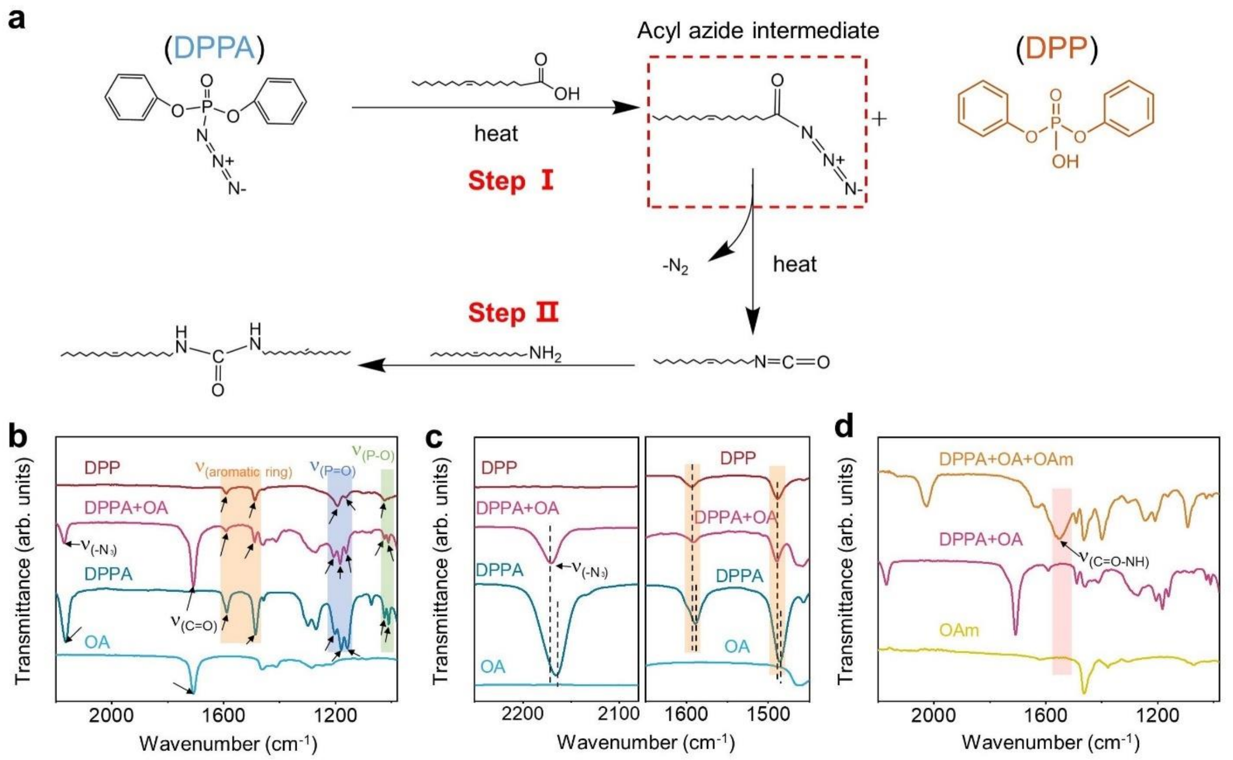

Supplementary Fig. 1 | Reaction processes between DPPA and precursor solvent. a, Schematic diagram of the reaction between DPPA and OA, followed by the subsequent reaction between the resulting acyl azide intermediate and OAm. **b, c, d,** Verification of the reaction process by FTIR spectra. (**b**) FTIR spectra of OA, DPPA, resultant of DPPA and OA, and DPP, respectively. The orange range indicates the stretching vibration of the aromatic ring, the blue range represents the stretching vibration of P=O bonds, and the green region indicates the stretching vibration of P-O bonds. (**c**) The enlarged FTIR spectra. The left depicts the enlarged infrared spectrum of the $-N_3$ stretching vibration, demonstrating a high-frequency shift after adding OA compared to pure DPPA. This shift is attributed to changes in adjacent functional groups, leading to an inductive effect. The right shows the enlarged infrared spectrum of the benzene ring, which also exhibits frequency variations. Furthermore, the peak positions of the benzene ring in DPPA+OA resultant align with those of pure DPP, indicating the formation of DPP from the reactants DPPA and OA. These results validate the reaction in Step I. (**d**) FTIR spectra of OAm, DPPA+OA, and DPPA+OA+OAm. Further addition of OAm results in a distinct peak at 1,554 cm^{-1}, corresponding to the vibration of the amide bond in the secondary amide, validating the process outlined in Step II.

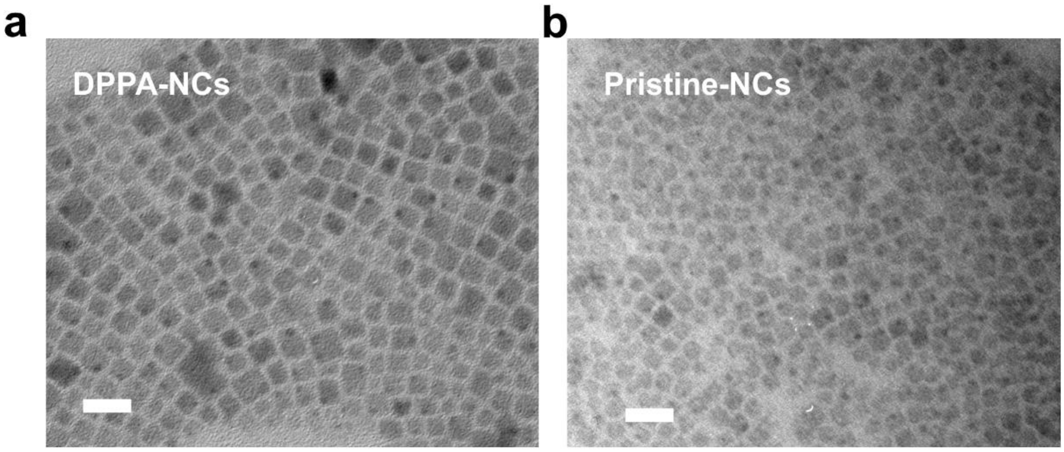

Supplementary Fig. 2 | TEM images. Scale bar: 20 nm. **a**, DPPA-NCs. **b**, Pristine-NCs.

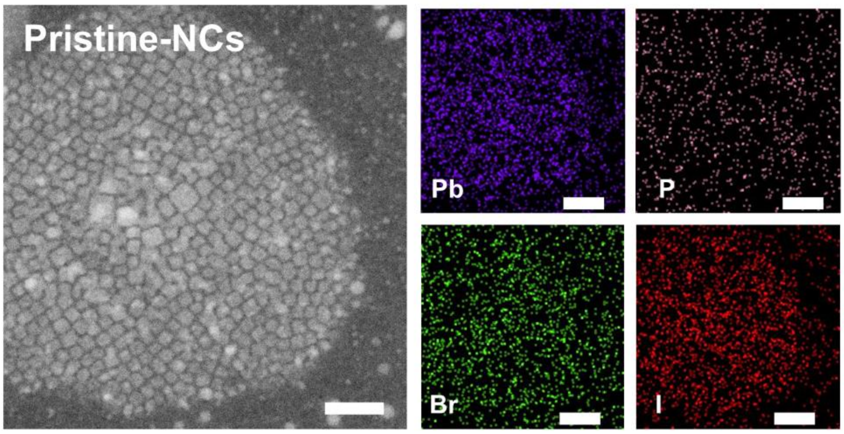

Supplementary Fig. 3 | HAADF-STEM image and corresponding elemental mapping images.

Scale bar: 50 nm.

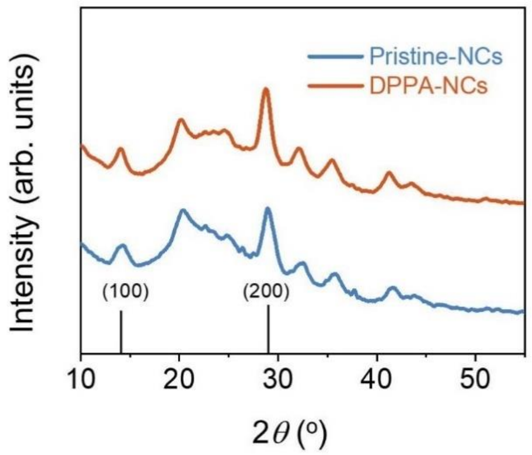

Supplementary Fig. 4 | XRD patterns of the pristine-NCs and DPPA-NCs.

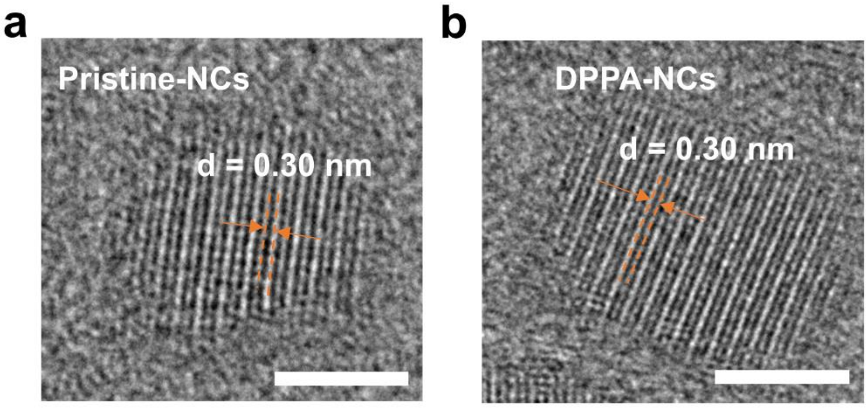

Supplementary Fig. 5 | HRTEM images. Scale bar: 5 nm. **a**, Pristine-NCs. **b**, DPPA-NCs.

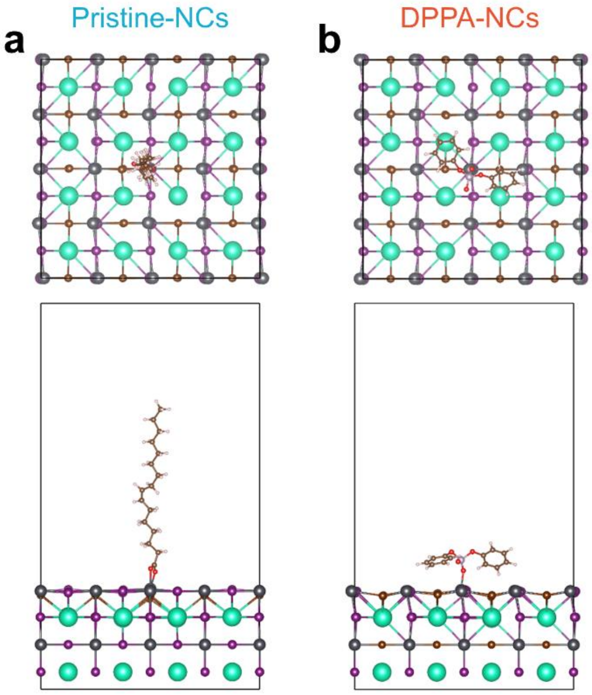

Supplementary Fig. 6 | Optimized crystal structures for the $CsPb(Br/I)_3$ slab models. a, Carboxylic ligands. **b**, DPP ligands. The top panels show the top view, while the bottom panels show the side view.

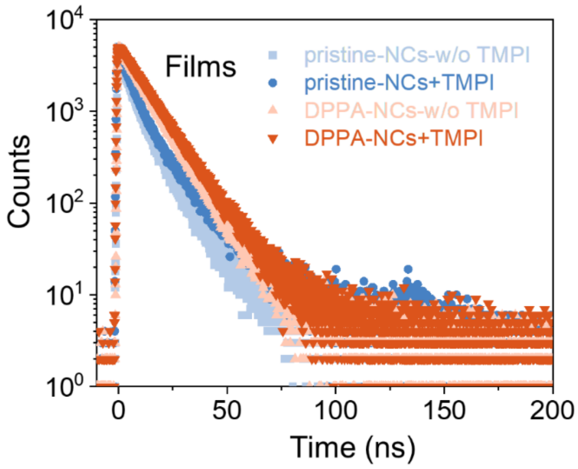

Supplementary Fig. 7 | Time-resolved photoluminescence decay curves of the NC films.

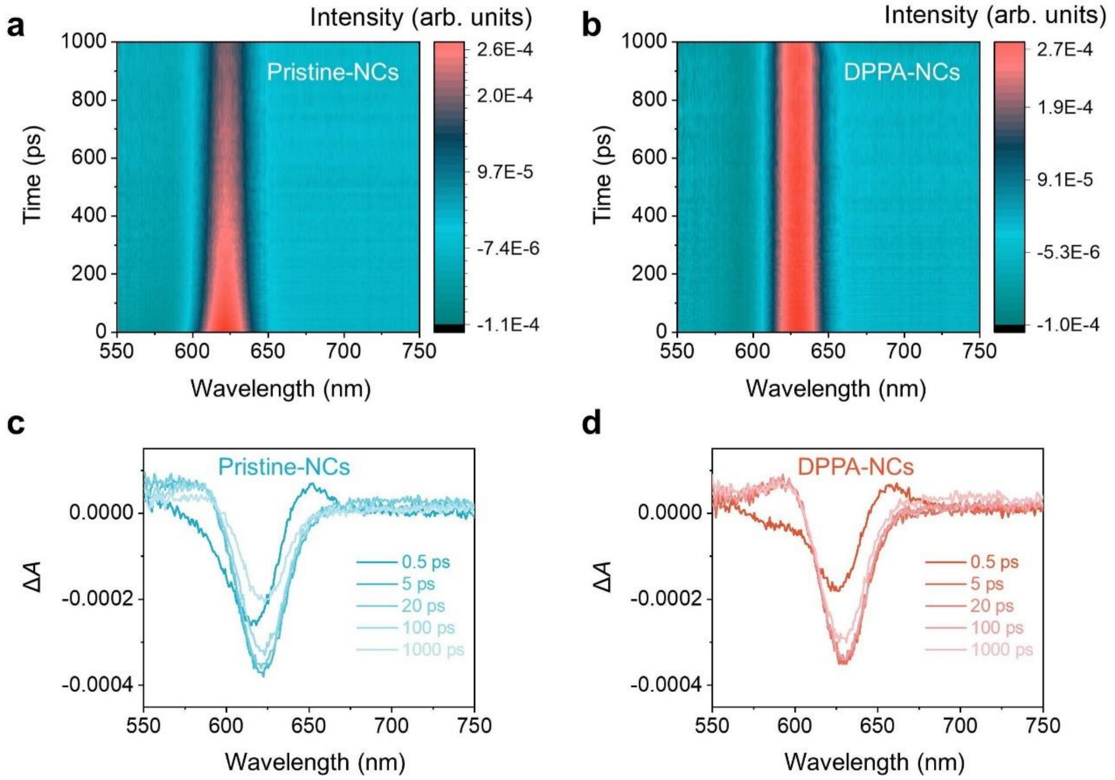

Supplementary Fig. 8 | TA measurements. a, b, Full-timescale pseudo-colour 2D representation of (**a**) pristine-NCs, and (**b**) DPPA-NCs, respectively. **c, d,** The fs-TA spectra of (**c**) pristine-NCs, and (**d**) DPPA-NCs at different delay times.

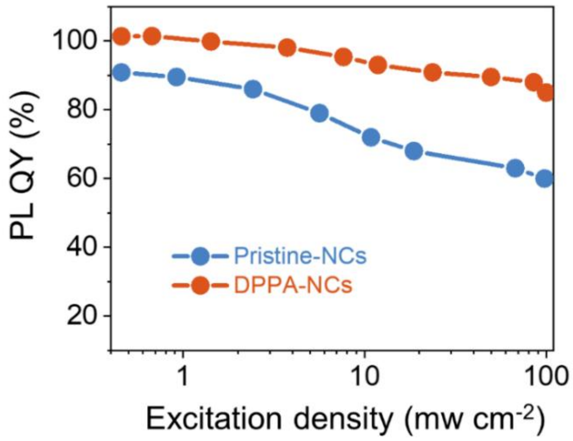

Supplementary Fig. 9 | Excitation intensity dependence of the PLQYs of the NC solution.

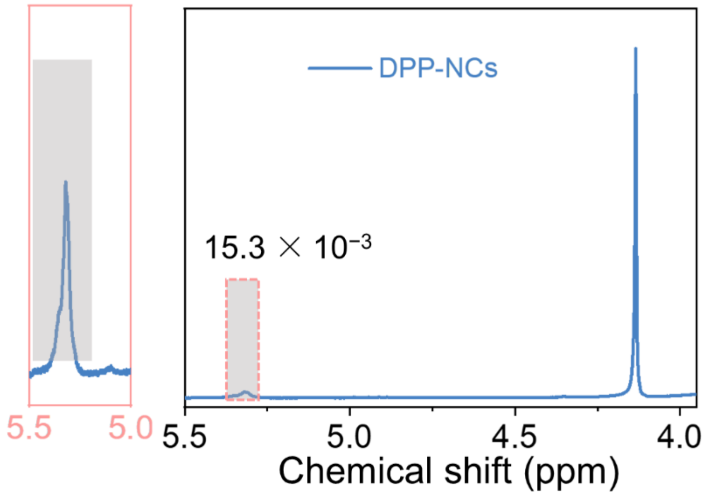

Supplementary Fig. 10 | The quantitative ^{1}H NMR spectrum of DPP-NCs. The left exhibits the enlarged spectra of the alkenyl region (δ=5.2–5.4 ppm). Ferrocene is used as the reference material.

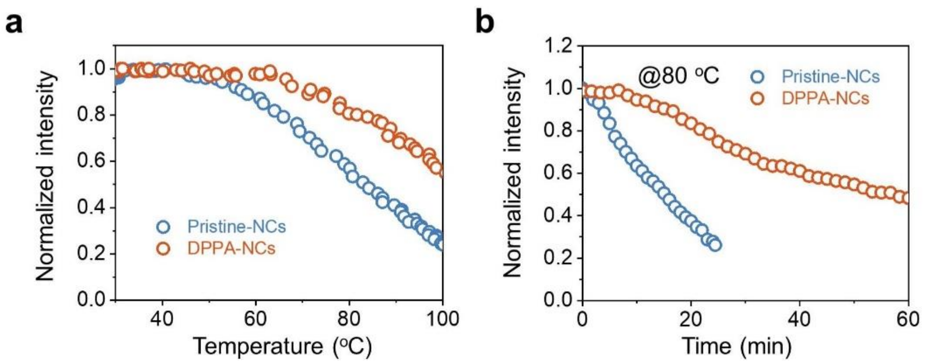

Supplementary Fig. 11 | Thermal stability of the NC films. a, The temperature-dependent normalized PL intensity trajectory of the NC films. **b**, The time-dependent PL intensity trajectory of the NC films at 80 °C.

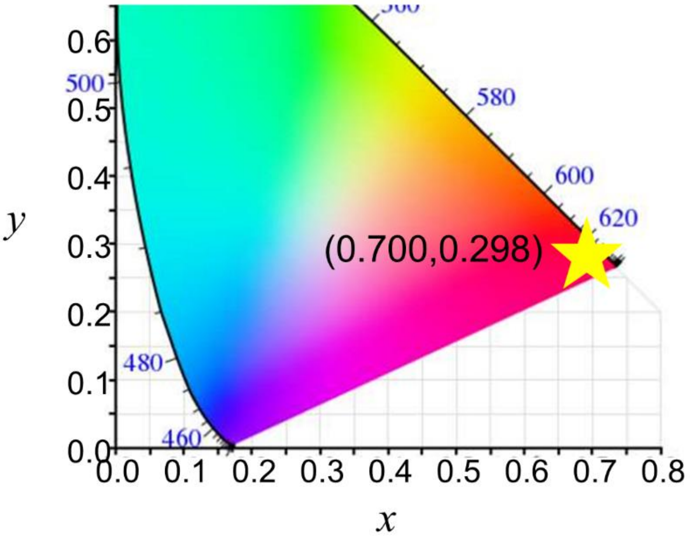

Supplementary Fig. 12 | CIE coordinate of PeLEDs based on DPPA-NCs.

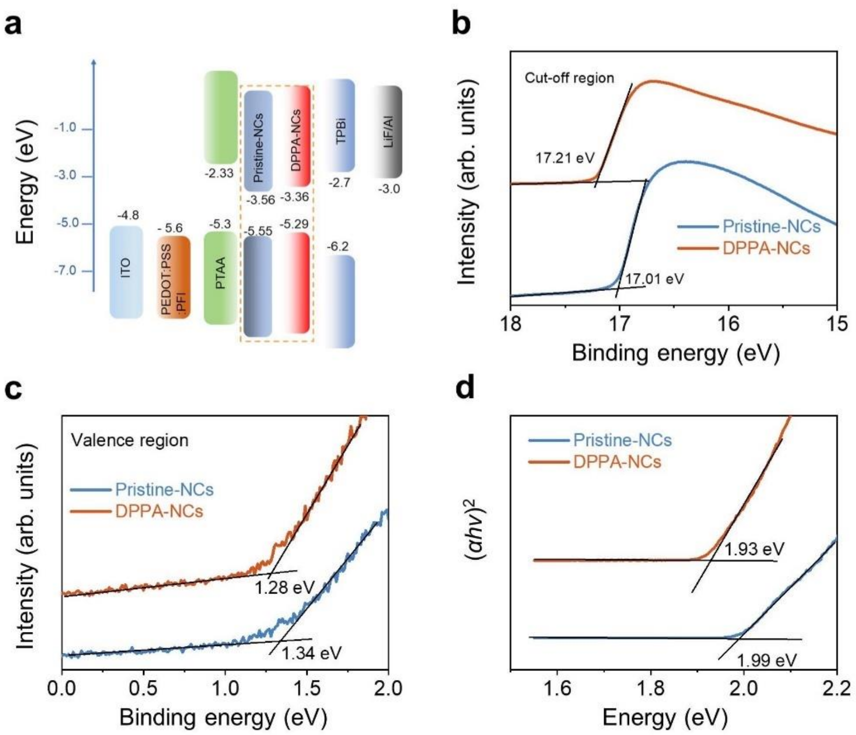

Supplementary Fig. 13 | The flat-band energy level diagram and UPS spectra. a, Devices energy-level diagram for each functional layer in the LEDs. **b**, Photoemission cutoff region. **c**, The valence-band-edge region. **d**, The plots of $(\alpha h\nu)^2$ versus the photon energy calculated from the absorption measurement. E_{VB} and E_{CB} are calculated from the following formula: $E_{\mathrm{VB}} = E_{\mathrm{cutoff}} + \Delta E$; $E_{\mathrm{CB}} = E_{\mathrm{VB}} - E_{\mathrm{optical\ bandgap}}$.

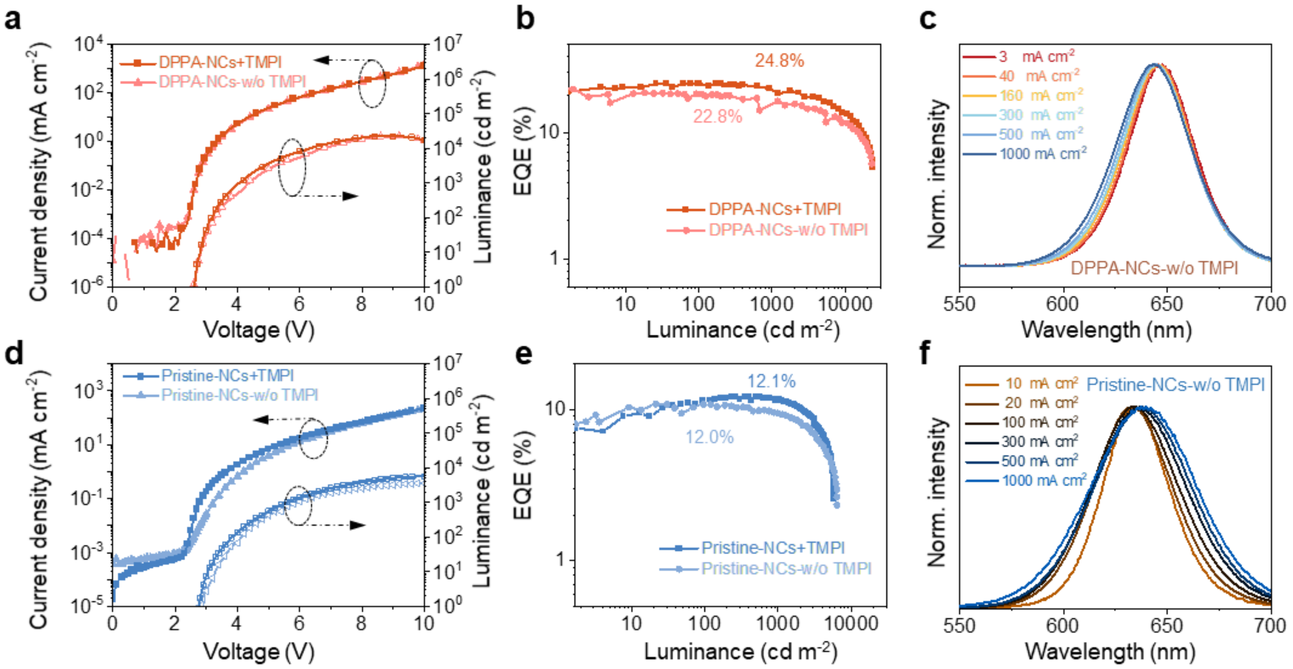

Supplementary Fig. 14 | Effect of TMPI on device performance. a-c, *J*-*V*-*L*, EQE−*L* characteristics, and EL spectra stability of the PeLEDs based on the DPPA-NCs+TMPI, and DPPA-NCs-w/o TMPI, respectively. **d-f,** *J*-*V*-*L***,** EQE−*L* characteristics, and EL spectra stability of the PeLEDs based on the pristine-NCs+TMPI, and pristine-NCs-w/o TMPI, respectively. The TMPI passivates the surface halide vacancy of the NCs, slightly improving the performance of the PeLEDs.

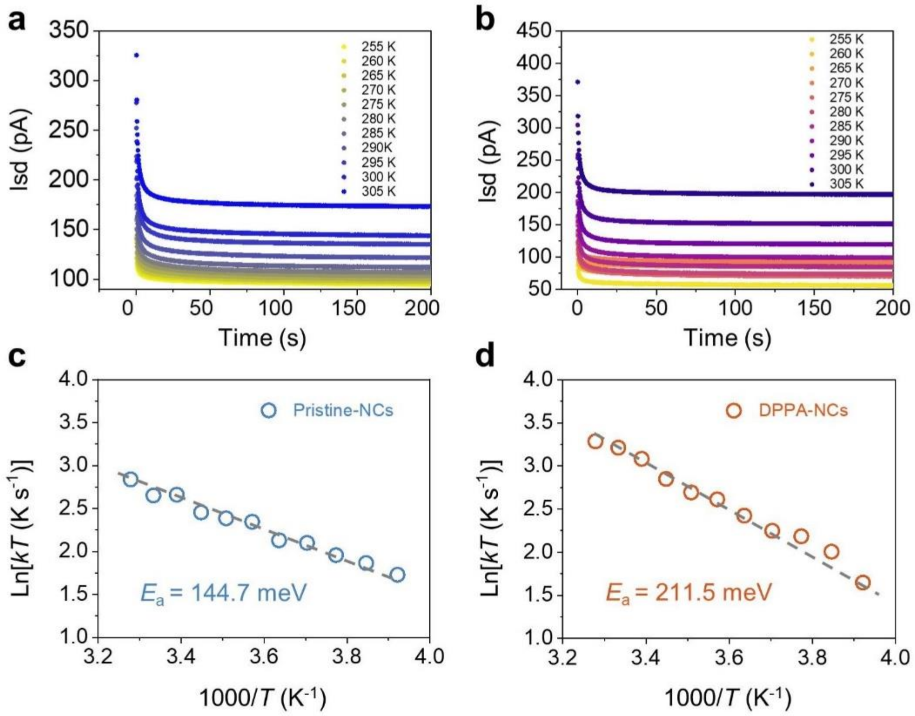

Supplementary Fig. 15 | Ion migration activation energy measurement. a, b, The time-dependent currents from 255 K to 305 K by applying bias at 12 V for the **(a)** pristine-NC film, and **(b)** DPPA-NC film, respectively. **c, d**, Temperature-dependent plots of ln(kT) versus 1,000/T for the **(c)** pristine-NC film, and **(d)** DPPA-NC film, respectively.

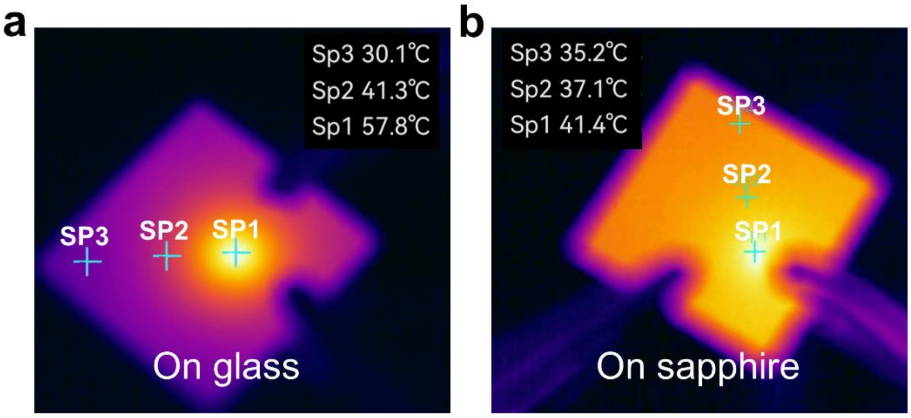

Supplementary Fig. 16 | Infrared-thermal-imaging images of the PeLEDs. a, On glass substrate. **b**, On sapphire substrate. Both of them are measured after operating at 1,000 mA cm^{-2} for 30 seconds. The temperatures at various positions on the substrate, relative to the luminous point, were also recorded.

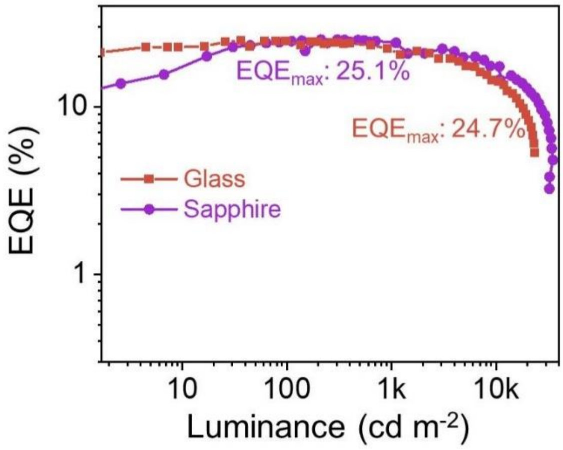

Supplementary Fig. 17 | EQE-*L* curves of DPPA-NC-based PeLEDs on the glass or sapphire substrate.

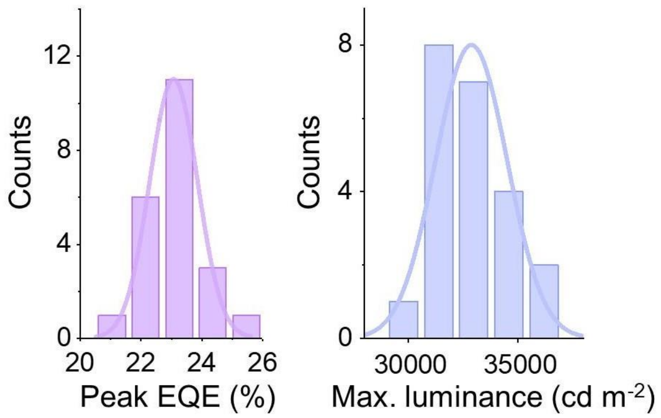

Supplementary Fig. 18 | Statistical distribution histograms of peak EQE and maximum luminance. The data was summarized from 22 DPPA-NC-based LEDs on sapphire substrate.

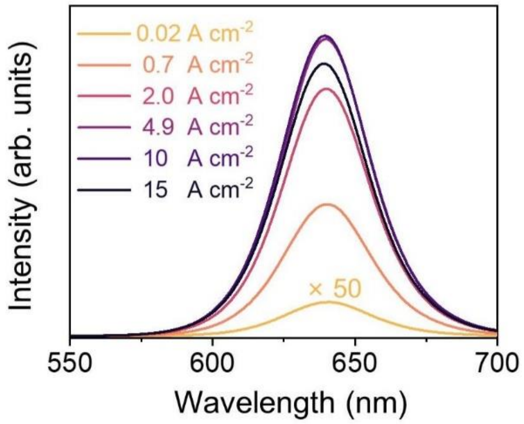

Supplementary Fig. 19 | EL spectra of the PeLEDs under different current densities. The PeLEDs were driven in pulsed mode operation with a duty cycle of 10% (50 Hz).

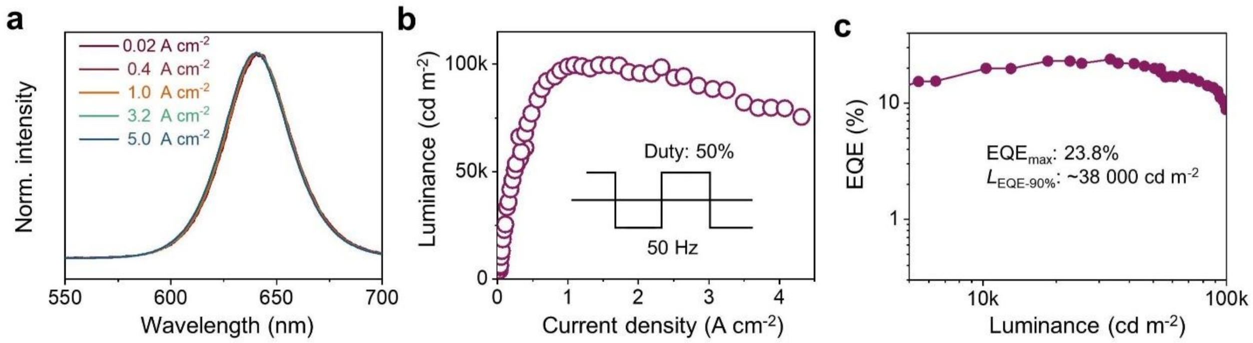

Supplementary Fig. 20 | Pulsed mode operation of PeLED on a sapphire substrate at 50 Hz with a duty cycle of 50%. **a**, Normalized EL spectra under different current densities. **b**, Luminance-current density curves. **c**, EQE-luminance curves.

Supplementary Table 1 | Summary of time-resolved PL exponential fitting parameters for NC solutions.

Solutions	f_1 (%)	τ_1 (ns)	f_2 (%)	τ_2 (ns)	τ_{av} (ns)	$\tau_{effective}$ (ns) [a]	PLQY (%)	$K_{nr}\times10^7$ (s^{-1})
**Pristine-NCs**	72.78	12.12	27.22	42.17	20.57	11.8	78%	1.06
**DPPA-NCs**	100	20.9			20.9	22.3	98%	0.096

[a] The effective decay time ($\tau_{effective}$) is extracted from the raw data, representing the time when the peak intensity drops to 1/e.

Supplementary Table 2 | Summary of time-resolved PL exponential fitting parameters for NC films.

Films	f_1 (%)	τ_1 (ns)	f_2 (%)	τ_2 (ns)	τ_{av} (ns)	PLQY (%)	$K_{nr}\times10^7$ (s^{-1})
**Pristine-NCs-w/o TMPI**	68.39	5.50	31.61	15.56	8.68	52%	5.53
**Pristine-NCs+TMPI**	70.16	6.44	29.8	20.71	10.69	58%	5.42
**DPPA-NCs-w/o TMPI**	98.53	10.87	1.47	59.75	11.6	87%	1.12
**DPPA-NCs+TMPI**	98.20	11.95	1.80	56.45	12.8	90%	0.78

Supplementary Table 3 | Summary of TA spectra fitting parameters.

NCs	A_1 (%)	τ_1 (ps)	A_2 (%)	τ_2 (ps)
**Pristine-NCs**	19.1	84.73	80.9	751.25
**DPPA-NCs**	100	542.26	/	/

Supplementary Table 4 | Summary of reported red PeLEDs with superior performance.

Samples	EL peak	EQE (%)	L_{max} (cd m^{-2})	Corresponding EQE at high L	T_{50} (min)[a]	Reference
$CsPbI_3$ QDs/NCs	633	20.1	4,932	10.5% @ 1,000 cd m^{-2}	150	1
	636	20.8	3,775	5.5% @ 1,000 cd m^{-2}	7.4	2
	637	26.1	2,511	16.0% @ 1,000 cd m^{-2}	450	3
	640	23	1,500	10.0% @ 900 cd m^{-2}	600	4
	642	28.5	4,140	15.1%@ 1,000 cd m^{-2}	786	*5*
	656	22.6	1,308	10.3% @ 1,000 cd m^{-2}	489	6
	668	22.5	955	4.8% @ 900 cd m^{-2}	624	7
	684	25.3	13,600	20% @ 1,000 cd m^{-2}	840	8
$CsPb(Br/I)_3$ NCs	635	22.8	12,910	10% @ 10,000 cd m^{-2}	63	9
	637	21.8	2,653	10.4% @ 935 cd m^{-2}	70	10
	640	23.5	1,510	6.0% @ 1,000 cd m^{-2}	97	11
	648	13.2	11,233	6.3%@10,000 cd m^{-2}	/	12
	653	22	7,000	20% @ 1,000 cd m^{-2}	T_{90}:760 h	*13*
	653	24.4	350	14.3%@200 cd m^{-2}	1200	14
	653	21.3	500	6.5% @ 300 cd m^{-2}	5	15
**This work**	**640**	**24.8**	**23,690**	**14.1% @ 10,000 cd m^{-2}**	**786**	**On glass**
		**24.2**	**35,120**	**17.6% @ 10,000 cd m^{-2}**	**1,200**	**On sapphire**
		**24.0**	**390,000**	**22% @ 40,000 cd m^{-2}**	/	**Pulsed**

[a] The T_{50} is summarized at the initial luminance of 100 cd m^{-2}.

Supplementary Table 5 | The fitting data from the current decay plots.

Temperature	Pristine-NCs		DPPA-NCs	
	τ_1 (s)	τ_2 (s)	τ_1 (s)	τ_2 (s)
255 K	0.71	45.20	0.73	49.09
260 K	0.71	40.24	0.61	34.95
265 K	0.76	37.43	0.67	29.82
270 K	0.80	33.06	0.75	28.51
275 K	0.83	32.66	0.77	24.34
280 K	0.87	26.8	0.75	20.54
285 K	0.92	26.22	0.81	19.29
290 K	0.95	24.89	0.79	16.78
295 K	0.94	20.62	0.76	13.52
300 K	0.93	21.15	0.75	12.09
305 K	0.86	17.84	0.72	11.43

Structural Characterization

The FT IR spectra were obtained by using a Thermo Fisher IS-50 spectrophotometer. The NC solution was dropped on KBr substrates for characterizations. XPS spectra were obtained on Thermo Scientific K-Alpha equipment in an ultrahigh vacuum chamber with a vacuum $<10^{-10}$ Torr. Ultraviolet photoelectron spectroscopy (UPS) was measured using an AXIS Supra (SHIMADZU) equipped with a He-I UV-light source (21.22 eV). The ^{1}H NMR spectra were recorded by a Brucker 400 MHz NMR spectrometer. TEM observations were conducted using a Hitachi HT-7700 microscope operated at 100 kV. HRTEM observations and high-angle annular dark-field scanning transmission electron microscopy (HAADF-STEM) images on the NCs were conducted using a JEMF2000 microscope operated at 200 kV. The TEM and HRTEM samples were prepared by dropping the suspension onto carbon-coated copper grids (300 mesh). Cross-sectional sample lamellae were cut and thinned down to electron transparency (~200 nm) with an FEI Helios Nanolab Dualbeam FIB/SEM following a standard protocol. The lamellae were then transferred directly into an FEI Osiris TEM operated at 200 kV. XRD was performed on Rigaku (SmartLab) operated at 40 keV and 40 mA with Cu Kα radiation (λ = 1.5406 Å).

Optical Characterization

UV–Vis absorption spectra were recorded by using an Agilent Cary 5000. The PL spectra were recorded by the Zolix system with a xenon flash lamp in the instrument as the source of excitation. The time-resolved fluorescence spectra were measured by the time-correlated single-photon counting technique. A commercially available TCSPC module (DCS900PC) was used to record the PL decay curves. The total instrument response function (IRF) is less than 150 ps. Transient absorption spectra were recorded using a femtosecond pump-probe spectroscopy setup. The fundamental output from a Light Conversion Pharos Yb: KGW laser (1,030 nm, 200 fs, 200 μJ/pulse, and 100 kHz repetition rate) was separated into two light beams. One beam was directed to a yttrium aluminum garnet (YAG) plate to produce a probe light with continuous spectra; the other was introduced to a Light Conversion Orpheus-HP optical parameter amplifier to generate a pump beam at 500 nm, which passed through a Newport M-ILS250HA optical delay line and a chopper working at 25 kHz driven by a Maxon EC motor, overlapping with the probe beam on the centre of the sample solution with a small angle. The transmitted probe light from the sample was then collected by a

Zolix λ200i spectrometer. The pump beam was blocked using a polarization plate. The instrument response function of the system is about 30 fs by measuring the probe light intensity change of a blank sample.

The PLQYs of the NCs were measured by a home-designed system[16, 17]. A system consisting of a xenon flash lamp, a QEPro spectrometer, and a home-designed integrating sphere was used to measure the absolute PLQYs. The wavelength and power density of excitation light were 450 nm and ~0.3 mW cm^{-2}, respectively. The PL intensity was integrated within the wavelength of 550 to 750 nm.

For excitation density-dependent measurements[18], the excitation light source was changed to a 405 nm continuous laser, which was modulated by a lock-in amplifier (SR830, Standard Research System). The PL intensity was recorded by a photodetector (Thorlabs PDA100A) that connected with the lock-in amplifier. A long-pass filter of 420 nm was placed right before the photodetector to filter the excitation light. 50% of the excitation light was irradiated on the sample through a beam splitter, and the rest was reflected on another photodetector to monitor the intensity of the excitation light. An attenuator was put before the excitation light source to control the power density on the samples ranging from 0.1-100 mW cm^{-2}. The PLQY$_x$, i.e., PLQY at a given power density (x mW cm^{-2}) of 405 nm excitation, can be determined by

$$\mathrm{PLQY}_x = \frac{\mathrm{PL}_x \times 0.3}{\mathrm{PL}_{0.3} \times x} \times \mathrm{PLQY}_{0.3} \tag{1}$$

where, PL_x, $PL_{0.3}$, $PLQY_{0.3}$ are the PL intensity at an excitation of x mW cm^{-2}, the PL intensity at an excitation of 0.3 mW cm^{-2}, and the absolute PLQY determined by integrating sphere system, respectively.

For the calculation of the nonradiative recombination rates, PL decays were fitted by the biexponential function:

$$A(t) = A_1 \exp\left(-\frac{t}{\tau_1}\right) + A_2 \exp\left(-\frac{t}{\tau_2}\right) + A_0 \tag{2}$$

where A_0, A_1, and A_2 are constants, t is time and τ_1, τ_2 are the decay times. The average PL lifetime (τ_{av}) was calculated as:

$$\tau_{\mathrm{av}} = \frac{A_1 {\tau_1}^2 + A_2 {\tau_2}^2}{A_1 \tau_1 + A_2 \tau_2} = f_1 \tau_1 + f_2 \tau_2 \tag{3}$$

Where:

$$f_1 = \frac{A_1\tau_1}{A_1\tau_1 + A_2\tau_2} \tag{4}$$

$$f_2 = \frac{A_2\tau_2}{A_1\tau_1 + A_2\tau_2} \tag{5}$$

Photoluminescence quantum yield in low-dimensional perovskite under low excitation power density can be described by the following formula:

$$\mathrm{PLQY} = \frac{k_\mathrm{r}}{k_\mathrm{nr} + k_\mathrm{r}} \tag{6}$$

The carrier decay time and carrier recombination rate (k_r, k_nr) have the following relationship:

$$k_\mathrm{nr} + k_\mathrm{r} = \frac{1}{\tau} \tag{7}$$

Thus, the radiative recombination rate:

$$k_\mathrm{r} = \frac{\mathrm{QY}}{\tau_\mathrm{av}} \tag{8}$$

and the nonradiative recombination rate[19]:

$$k_\mathrm{nr} = \frac{1 - \mathrm{QY}}{\tau_\mathrm{av}} \tag{9}$$

Ion migration activation energy measurement

The decay in the temperature-dependent temporal response can reflect the kinetics of ionic movement. The temperature-dependent ionic conductivity σ was addressed as[20]:

$$\sigma(T) = \sigma_0 \exp\left(-\frac{E_\mathrm{a}}{k_B T}\right) \tag{10}$$

Where E_a is the activation energy for ion transport, k_B is the Boltzmann constant. The decay rate ($k = \tau^{-1}$) of the current decay represents the ionic transport dynamics and is proportional to the ionic conductivity. Therefore, the temperature-dependent decay rates can be used to obtain the thermal active energy for ion migration.

The ion migration activation energy measurement was carried out on the device with a structure of glass/NCs/Au. The NC solution (~15 mg $\mathrm{mL^{-1}}$ in octane) was spun onto the glass at 4000 rpm for 45 s. Then the Au interdigital electrode (80 nm) was deposited onto the NC film by thermal evaporation under a high vacuum ($< 5 \times 10^{-4}$ Pa). The samples were placed on a liquid nitrogen thermostat, and Keithley 2635B was used to detect the current signal. The current decay curves were obtained at 12 V and fitted by a double exponential function. The τ_1 is independent of temperature, which relates to the equipment response. The τ_2 represents the time constant of ion migration and is used for calculating the activation energy**.** The ion migration activation $\mathrm{E_a}$ was calculated as the

slope of ln(kT)-1/T using the relation

$$\mathrm{Ln}(kT) = \mathrm{C} - \frac{E_{\mathrm{a}}}{\mathrm{K_B}T} \tag{11}$$

where k can be obtained by $k = \tau^{-1}$ using the time constant τ_2 at different temperatures from 255 K to 305 K.

DFT calculations

We utilized the first-principles tool, the Vienna Ab initio Simulation Package (VASP)[21, 22], for all density functional theory (DFT) calculations within the generalized gradient approximation (GGA) employing the Perdew-Burke-Ernzerhof (PBE)[23] formulation. Projected augmented wave (PAW) potentials[24, 25] were chosen to describe the ionic cores, incorporating valence electrons using a plane wave basis set with a kinetic energy cutoff of 450 eV. Partial occupancies of the Kohn−Sham orbitals were permitted using the Gaussian smearing method with a width of 0.05 eV. For geometry and lattice size optimization, Brillouin zone integration was conducted with 1×1×1 Γ-centered k-point sampling[26]. Self-consistent calculations applied a convergence energy threshold of 10^{-5} eV. Equilibrium geometries and lattice constants were optimized with a maximum stress on each atom within 0.02 eV $Å^{-1}$. The 35 Å vacuum layer was typically added to the surface to eliminate artificial interactions between periodic images, with the bottom 3 atom layers fixed. Weak interactions were described using the DFT+D3 method employing empirical correction in Grimme's scheme[27, 28]. The spin polarization method was adopted to characterize the magnetic system. Furthermore, the crystal orbital Hamilton population (COHP) was calculated using the Lobster too[29-32]

Supplementary references

1. Guo, J. *et al.* Entropy-Driven Strongly Confined Low-Toxicity Pure-Red Perovskite Quantum Dots for Spectrally Stable Light-Emitting Diodes. *Nano Lett.* **24**, 417-423 (2024).
2. Xie, M. *et al.* High-Efficiency Pure-Red Perovskite Quantum-Dot Light-Emitting Diodes. *Nano Lett.* **22**, 8266-8273 (2022).
3. Feng, Y. *et al.* Nucleophilic Reaction-Enabled Chloride Modification on CsPbI3 Quantum Dots for Pure Red Light-Emitting Diodes with Efficiency Exceeding 26 %. *Angew. Chem. Int. Ed.* **n/a**, e202318777 (2024).
4. Wang, Y. *et al.* All-Inorganic Quantum-Dot LEDs Based on a Phase-Stabilized α-$CsPbI_3$ Perovskite. *Angew. Chem. Int. Ed.* **60**, 16164-16170 (2021).
5. Li, H. *et al.* Nanosurface-reconstructed perovskite for highly efficient and stable active-matrix light-emitting diode display. *Nat. Nanotechnol.* **19**, 638-645 (2024).
6. Zhao, F. *et al.* Iodotrimethylsilane as a Reactive Ligand for Surface Etching and Passivation of Perovskite Nanocrystals toward Efficient Pure-red to Deep-red LEDs. *Angew. Chem. Int. Ed.* **62**, e202311089 (2023).
7. Ebe, H. *et al.* Guanidium iodide treatment of size-controlled CsPbI3 quantum dots for stable crystal phase and highly efficient red LEDs. *Chem. Eng. J.* **471**, 144578 (2023).
8. Guo, J. *et al.* Highly Stable and Efficient Light-Emitting Diodes Based on Orthorhombic γ-CsPbI3 Nanocrystals. *ACS Nano* **17**, 9290-9301 (2023).
9. Zhang, J. *et al.* Ligand-induced Cation-Π Interactions Enable High-Efficiency, Bright And Spectrally Stable Rec. 2020 Pure-Red Perovskite Light-Emitting Diodes. *Adv. Mater.* **35**, 2303938 (2023).
10. Xie, M. *et al.* Suppressing Ion Migration of Mixed-Halide Perovskite Quantum Dots for High Efficiency Pure-Red Light-Emitting Diodes. *Adv. Funct. Mater.* **33**, 2300116 (2023).
11. Zhang, J. *et al.* A Multifunctional" halide-Equivalent" Anion Enabling Efficient $CsPb (Br/I)_3$ Nanocrystals Pure-Red Light-Emitting Diodes with External Quantum Efficiency Exceeding 23. *Adv. Mater.* **35**, 2209002 (2022).
12. Shen, X. *et al.* Bright and Efficient Pure Red Perovskite Nanocrystals Light‐Emitting Devices via In Situ Modification. *Adv. Funct. Mater.* **32**, 2110048 (2022).

13. Wang, Y-K. *et al.* Long-range order enabled stability in quantum dot light-emitting diodes. *Nature* **629**, 586-591 (2024).
14. Wang, Y-K. *et al.* In-situ inorganic ligand replenishment enables bandgap stability in mixed-halide perovskite quantum dot solids. *Adv. Mater.* **34**, 2200854 (2022).
15. Chiba, T. *et al.* Anion-exchange red perovskite quantum dots with ammonium iodine salts for highly efficient light-emitting devices. *Nat. Photonics* **12**, 681-687 (2018).
16. de Mello, JC., Wittmann, HF. & Friend, RH. An improved experimental determination of external photoluminescence quantum efficiency. *Adv. Mater.* **9**, 230-232 (1997).
17. Zhang, Z. *et al.* High-Performance, Solution-Processed, and Insulating-Layer-Free Light-Emitting Diodes Based on Colloidal Quantum Dots. *Adv. Mater.* **30**, 1801387 (2018).
18. Liu, Y. *et al.* Efficient blue light-emitting diodes based on quantum-confined bromide perovskite nanostructures. *Nat. Photonics* **13**, 760-764 (2019).
19. Yang, S. *et al.* Electron Delocalization in CsPbI3 Quantum Dots Enables Efficient Light-Emitting Diodes with Improved Efficiency Roll-Off. *Adv. Opt. Mater.*, 2200189 (2022).
20. Zhang, B-B. *et al.* Defect proliferation in $CsPbBr_3$ crystal induced by ion migration. *Appl. Phys. Lett.* **116**, 063505 (2020).
21. Kresse, G. & Furthmüller, J. Efficiency of ab-initio total energy calculations for metals and semiconductors using a plane-wave basis set. *Comput. Mater. Sci.* **6**, 15-50 (1996).
22. Kresse, G. & Furthmüller, J. Efficient iterative schemes for ab initio total-energy calculations using a plane-wave basis set. *PhRvB* **54**, 11169-11186 (1996).
23. Perdew, JP., Burke, K. & Ernzerhof, M. Generalized Gradient Approximation Made Simple. *Phys. Rev. Lett.* **77**, 3865-3868 (1996).
24. Blöchl, PE. Projector augmented-wave method. *PhRvB* **50**, 17953-17979 (1994).
25. Kresse, G. & Joubert, D. From ultrasoft pseudopotentials to the projector augmented-wave method. *PhRvB* **59**, 1758-1775 (1999).
26. Pack, JD. & Monkhorst, HJ. "Special points for Brillouin-zone integrations"---a reply. *PhRvB* **16**, 1748-1749 (1977).
27. Grimme, S., Antony, J., Ehrlich, S. & Krieg, H. A consistent and accurate ab initio parametrization of density functional dispersion correction (DFT-D) for the 94 elements H-Pu.

J. Chem. Phys. **132**, 154104 (2010).

28. Grimme, S., Ehrlich, S. & Goerigk, L. Effect of the damping function in dispersion corrected density functional theory. *J. Comput. Chem.* **32**, 1456-1465 (2011).
29. Dronskowski, R. & Bloechl, PE. Crystal orbital Hamilton populations (COHP): energy-resolved visualization of chemical bonding in solids based on density-functional calculations. *J. Phys. Chem. C* **97**, 8617-8624 (1993).
30. Deringer, VL., Tchougréeff, AL. & Dronskowski, R. Crystal Orbital Hamilton Population (COHP) Analysis As Projected from Plane-Wave Basis Sets. *J. Phys. Chem. A* **115**, 5461-5466 (2011).
31. Maintz, S., Deringer, VL., Tchougréeff, AL. & Dronskowski, R. LOBSTER: A tool to extract chemical bonding from plane-wave based DFT. *J. Comput. Chem.* **37**, 1030-1035 (2016).
32. Nelson, R., Ertural, C., George, J., Deringer, VL., Hautier, G. & Dronskowski, R. LOBSTER: Local orbital projections, atomic charges, and chemical-bonding analysis from projector-augmented-wave-based density-functional theory. *J. Comput. Chem.* **41**, 1931-1940 (2020).
